# Fatigue in breast cancer patients on chemotherapy: a cross-sectional study exploring clinical, biological, and genetic factors

**DOI:** 10.1186/s12885-021-09072-0

**Published:** 2022-01-03

**Authors:** Aline Hajj, Rami Chamoun, Pascale Salameh, Rita Khoury, Roula Hachem, Hala Sacre, Georges Chahine, Joseph Kattan, Lydia Rabbaa Khabbaz

**Affiliations:** 1 Faculty of Pharmacy, Saint-Joseph University of Beirut, Beirut, Lebanon; 2 Laboratoire de Pharmacologie, Pharmacie Clinique et Contrôle de Qualité des Médicaments, Faculty of Pharmacy, Saint-Joseph University of Beirut, Beirut, Lebanon; 3 INSPECT-LB (Institut National de Santé Publique, d’Épidémiologie Clinique et de Toxicologie-Liban), Beirut, Lebanon; 4 Faculty of Pharmacy, Lebanese University, Beirut, Lebanon; 5 University of Nicosia Medical School, Nicosia, Cyprus; 6 Department of Hemato-Oncology, Hôtel-Dieu de France Hospital, Faculty of Medicine, Saint-Joseph University of Beirut, Beirut, Lebanon

**Keywords:** Breast cancer, Chemotherapy, *CLOCK*, *DRD2*, Fatigue, Pharmacogenetics

## Abstract

**Background:**

Cancer-related fatigue (CRF) is one of the most common and distressing complaints reported by cancer patients during chemotherapy considerably impacting all aspects of a patient’s life (physical, psychosocial, professional, and socioeconomic). The aim of this study was to assess the severity of cancer-related fatigue in a group of breast cancer patients undergoing chemotherapy and explore the association between fatigue scores and sociodemographic, clinical, biological, psychiatric, and genetic factors.

**Methods:**

A cross-sectional pilot study carried out at the oncology outpatient unit of Hôtel-Dieu de France University Hospital recruited 67 breast cancer patients undergoing chemotherapy between November 2017 and June 2019 to evaluate fatigue using the EORTC QLQ-C30 scale (European Organization for the Research and Treatment of Cancer Quality of Life Questionnaire). Genotyping for seven gene polymorphisms (*COMT*, *DRD2*, *OPRM1*, *CLOCK*, *PER2*, *CRY2*, *ABCB1*) was performed using the Lightcycler^®^ (Roche).

**Results:**

The prevalence of fatigue was 46.3%. Multivariable analysis taking the fatigue score as the dependent variable showed that a higher number of cycles and a lower hemoglobin level were significantly associated with higher odds of exhibiting fatigue. Moreover, having at least one C allele for *DRD2* SNP (vs. TT) was significantly associated with a 4.09 higher odds of expressing fatigue compared to TT patients. Finally, patients with at least one C allele for *CLOCK* SNP tended to display higher fatigue levels than TT patients.

**Conclusions:**

Our study showed that anemic breast cancer patients with a high number of chemotherapy cycles and those carrying at least one C allele for *DRD2* and *CLOCK* SNPs are at greater risk of exhibiting fatigue. Since no previous research has reported such genetic results, future studies are necessary to confirm our findings.

## Background

According to the 2020 global cancer burden, female breast cancer ranked among the most commonly diagnosed cancer [1]. In this context, chemotherapy and radiotherapy remain the mainstay of cancer treatment. Thus, every year more than 2.3 million women encounter numerous side effects with devastating consequences on their health [1, 2]. Cancer-related fatigue (CRF) is one of the most common and distressing complaints reported by cancer patients during chemotherapy [3, 4]. Described as a multidimensional physical and/or mental tiredness or exhaustion that interferes with motivation and usual functioning [5], CRF results in substantial impairment of health-related quality of life (HRQoL) in breast cancer survivors [2, 6, 7]. Studies have shown that fatigue experienced by cancer patients undergoing chemotherapy is persistent and may remain beyond the chemotherapy session, considerably impacting all aspects of a patient’s life: physical, psychosocial, professional, and socioeconomic [8].

Despite its burden and relatively high prevalence among breast cancer patients (ranging from 60 to 90%) [9], CRF remains underestimated and mistreated, and little is known about the underlying risk factors. Understanding the contributing factors would allow the implementation of adequate targeted interventions for better management and quality of care [2, 7, 10]. Several hypotheses have been suggested to identify the predisposing factors to higher sensitivity for tiredness, including neurobiological dysfunctions (alterations in the hypothalamic-pituitary-adrenal (HPA) axis [11] and the autonomic nervous system responsiveness [12, 13]), pro-inflammatory cytokines and cellular immune system dysregulations [14, 15], psychological disorders such as depression, anxiety, and sleep disorders [16, 17], cancer treatments (e.g., regimen type, chemotherapy agents, doses) [18], in addition to other factors related to physical adaptability, pain [19, 20], or genetic predisposition [21–23].

Regarding the genetic factors, most studies among cancer patients evaluated the potential contribution of single nucleotide polymorphisms (SNPs) in the immune and inflammatory pathways, such as pro-inflammatory cytokines IL-1b, IL-6, and TNF-α [21]. However, these studies yielded conflicting results, possibly due to cancer itself; treatments could trigger a cytokine storm that may differ according to the type of cancer, disease stage, and regimen (all of which induce an epigenetic regulation) [21]. Therefore, we hypothesized that other genetic factors might also have a contributing role but have been scarcely explored with CRF. These include genes involved in different pathways, such as dopamine neurotransmission, opioid circuits, circadian rhythms, in addition to genes affecting the transport of xenobiotics to the central nervous system (chemotherapy drugs or pro-inflammatory mediators) [18, 21, 24–26].

Regarding the dopamine pathway, this study will explore the eventual correlation between SNPs in genes encoding the dopamine receptor D2 (*DRD2*) and the metabolic enzyme catechol-O-methyl transferase (COMT). Indeed, Miller et al. have previously reported impaired dopaminergic striatal functioning in individuals with chronic fatigue syndrome [24]. Studies have shown that the SNP c.957C > T (rs6277) in *DRD2* affects the striatal D2 receptor availability, leading to a decreased *DRD2* mRNA stability and receptor synthesis, consequently altering dopamine’s signal transduction [25]. As for *COMT*, the studied SNP p.Val158Met (p.V158M; rs4680) leads to 3-to-4 times lower COMT enzymatic activity [26]; patients carrying the Met variant allele exhibit higher levels of catecholamines, such as epinephrine, which promotes a higher pain sensitivity by stimulating β2-adrenergic receptors in the central and peripheral nervous systems [27]. A study had demonstrated that breast cancer patients with Met/Met genotype exhibit higher fatigue and pain sensitivity after surgery (mastectomy or quadrantectomy), stating that higher pain intensity can predispose to increased CRF [22].

In the context of pain regulation, *OPRM1* represents a crucial candidate gene for CRF. It encodes for the μ-opioid receptor (MOR) that regulates the analgesic response to pain and plays an essential role in the rewarding system [28]. The SNP c.118A > G (rs1799971) is the most explored polymorphism in *OPRM1*, leading to an asparagine-to-aspartic acid substitution at residue 40 (p.Asn40Asp), with a reduced affinity for endogenous opioids. Patients who carry at least one G variant allele exhibit higher pain levels than AA patients [29]. Consequently, acknowledging that increased pain sensitivity is associated with a dysregulation in pro-inflammatory cytokines, it is hypothesized that an alteration in the opioid system could potentially contribute to CRF in breast cancer patients [30].

Furthermore, owing to the fact fatigue is biologically regulated by a sleep/wake homeostatic process [31, 32], our study evaluated three of the circadian rhythm regulation genes: the Circadian Locomotor Output Cycles Kaput *CLOCK* gene (SNP c.3111 T > C; rs1801260), the Period 2 (*PER2*) gene (rs934945; G > A), and the Cryptochrome circadian Regulator 2 (*CRY2*) gene (rs10838524; G > A). Studies exploring these polymorphisms in CRF are scarce, and none have been performed in breast cancer patients. Research had found that the minor allele A of *PER2* rs934945 was associated with lower odds of fatigue in patients with gliomas [23]. Other studies in non-cancer patients have reported an association between the C-allele in the SNP rs1801260 of *CLOCK* with eveningness that could contribute to a lower morning physical activity [33, 34].

Finally, regarding the drug efflux transporters, our study will examine the SNP rs1045642 (c.3435 T > C) in *ABCB1,* the gene encoding the P-glycoprotein (P-gp). This SNP has been associated with functional changes in mRNA stability and P-gp expression. Patients with the variant T allele could potentially report more fatigue than those who carry the wild-type genotype due to a lower efflux at the blood-brain barrier (BBB) level and higher drug concentration in the brain, especially that almost all cytotoxic drugs for breast cancer are substrates of P-gp [35, 36]. Based on this hypothesis, various studies have previously demonstrated a significant association between CRF and three gene polymorphisms in *ABCB1*: c.2677G > A/T (rs2032582) in breast cancer patients receiving docetaxel [37], and c.1236C > T (rs1128503) and c.3435C > T (rs1045642) in patients with gynecologic cancers receiving paclitaxel and carboplatin [38]. However, no previous studies have identified a correlation between our studied SNP and CRF.

Therefore, this pilot study aimed to assess the severity of cancer-related fatigue in a group of breast cancer patients undergoing chemotherapy and explore the association between fatigue scores and clinical, biological, sociodemographic, psychiatric, and genetic factors.

## Methods

### Study design

This cross-sectional pilot study evaluated the effect of sociodemographic, clinical, biological, psychiatric, and genetic factors on fatigue among breast cancer patients undergoing chemotherapy at the oncology outpatient unit of Hôtel-Dieu de France (HDF) University Hospital between November 2017 and June 2019.

### Ethics approval

The HDF ethics committee approved the study (reference number: CEHDF1016, July 2017), and all patients signed a written consent prior to inclusion. All methods were carried out in accordance with relevant guidelines and regulations.

### Patient’s sociodemographic and clinical information

Included patients were women aged 18 and above, with a primary diagnosis of breast cancer, and admitted to the outpatient oncology unit at HDF for intravenous chemotherapy every 21 days (random cycle out of a maximum of 10 cycles).

Non-inclusion criteria consisted of patients with relapsed breast cancer/other types of cancer, receiving adjuvant hormone therapy at the moment of the evaluation, having brain metastasis, or any other medical/surgical CNS disorders that may affect their ability to complete the questionnaires or be assessed clinically [39–41].

Three trained pharmacists collected sociodemographic and clinical information from medical records or through interviews with the patients: age, gender, weight, and height (to calculate the body mass index, BMI), Body Surface Area (BSA, calculated using the Mosteller formula) [42, 43] ethnicity/nationality, marital status, education level, socioeconomic level, comorbidities (e.g., diabetes, hypertension, dyslipidemia), alcohol consumption, smoking, medical history of allergic reactions, and medications used other than chemotherapy. They also recorded biological values at baseline, including creatinine levels (to calculate the creatinine clearance ClCr using Cockcroft-Gault formula [44, 45]) and complete blood count (CBC), in addition to cancer-related data: metastases, the number of chemotherapy cycles, chemotherapy regimen (medications and doses/m^2^).

On the first day of admission to the outpatient oncology unit to receive chemotherapy (random cycle, recorded as the actual chemotherapy cycle number), patients completed a self-reported questionnaire that included several validated scales to evaluate fatigue, sleep, anxiety, depression, and pain. Pharmacists assisted them in completing it and made sure they answered all questions.

### Outcomes and clinical assessments

#### Fatigue

The primary outcome was cancer-related fatigue. Fatigue was evaluated using three questions from the EORTC QLQ-C30 scale (European Organization for the Research and Treatment of Cancer Quality of Life Questionnaire), a 30-item instrument that measures the quality of life (QOL) in cancer patients in three main domains: global health status, functional status, and cancer-related symptom status. The questions rated on a 4-point Likert scale from 0 (not at all) to 4 (very much) were: QLQ C10: “Do you need rest?”; QLQ C12: “Did you feel weak?” and QLQ C18: “Were you tired?” [46]. The raw value obtained for each participant was then transformed according to the EORTC QLQ-C30 scoring manual into a score ranging from 0 to 100, with higher scores indicating worse fatigue and thus lower QOL.

#### Pain

The visual analogue scale (VAS) was used to evaluate pain. This subjective tool enables patients to measure disease-related pain on a line ranging from 0 (no pain) to 10 (extreme pain) [47].

#### Sleep

Two screening tools were used to evaluate sleep disorders:

- The Insomnia Severity Index (ISI) is a 7-item scale designed to assess the perceived severity of insomnia during the past 2 weeks. Items are rated on a 5-point Likert scale. The total score ranges from 0 to 28, with higher scores indicating more severe insomnia [48].
- The Pittsburgh Sleep Quality Index (PSQI) is a 19-item tool developed to measure seven domains over the past month: subjective quality of sleep, sleep latency, sleep duration, sleep efficiency, sleep disorders, sleep medication, and daytime dysfunction. The seven sub-scores are rated from 0 (no difficulty) to 3 (severe difficulty) and yield a total score ranging from 0 to 21. Higher scores indicate worse sleep quality [49].

#### Anxiety and depression

The self-report Hospital Anxiety and Depression Scale (HADS) was used to explore the level of anxiety (HADS-A) and depression (HADS-D) during the previous week. Symptoms were reported on a scale from 0 (not at all) to 3 (most of the time) [50]. Higher scores defined higher levels of anxiety/depression.

### Data and statistical analysis

Three pharmacists collected the data and performed data entry. The SPSS software version 25.0 was used for statistical analysis, performed by one of the authors on de-identified data. Descriptive statistics were calculated for all variables in the study as means and standard deviations for continuous measures and counts and percentages for categorical variables. As the dependent variable was not normally distributed and the sample size was small (*n* = 67), non-parametric tests were used: the Mann-Whitney test to compare means between two groups, Kruskal-Wallis test to compare three or more groups (with post hoc analysis), and Spearman correlation to correlate between continuous, ordinal, or count variables. DNA sampling as well as genotyping assays were performed as previously prescribed [51]. The genotype alleles were taken once as three categories, then combined and checked for any significant association with the dependent variable. Variables that showed *p* < 0.1 in the bivariate analysis were taken as independent variables in the multivariable analyses to reduce confounding factors. In all cases, a value of *p* ≤ 0.05 was considered statistically significant.

As for multivariable analyses, logistic regression models were used after dichotomization of the fatigue scale: participants with scores > 39 were considered to have fatigue (39 is the defined threshold for clinical importance (TCIs) for the EORTC QLQ-C30 Computer Adaptive Testing Core measure) [52, 53]. Independent variables groups were subsequently included in the regression models, using the ENTER method: cycle number, cancer treatment, and biological measures. The results related to 7 genes were also used and added to the model with variables that showed a *p* < 0.10; the last step represented a global model of genetic, sociodemographic, and clinically related factors.

## Results

A total of 67 women with breast cancer were included in the study (mean age = 56.22 ± 11.96; mean BSA = 1.76 ± 0.17). Most of our patients were married (85,5%), with a secondary level of education. Almost 46% had a clinically significant fatigue, with a mean fatigue score of 42.12 ± 32.10 (as evaluated by the EORTC QLQ-C30) (Table 1).

**Table 1:** Sociodemographic and baseline characteristics (*N* = 67)

		**Frequency (%)**
Gender	Female	67 (100%)
Nationality	Lebanese	60 (89.6%)
	Syrian	5 (7.5%)
	Other	2 (3%)
Marital status	Single	8 (11.9%)
	Married	58 (86.6%)
	Widowed	1 (1.5%)
Level of education^a^	Elementary	9 (13.8%)
	Secondary	41 (63.1%)
	University	15 (23.1%)
Profession/Work	No	45 (67.2%)
	Yes	22 (32.8.%)
	**Mean ± Standard Deviation (SD)**	
Age (years)	56.22 ± 11.96	
Body Mass Index (BMI; Kg/m^2^)	26.06 ± 3.79	
Body Surface Area (BSA; m^2^)	1.76 ± 0.17	
Pain VAS score	1.27 ± 2.08	
**Fatigue Score**	42.12 ± 32.10	
▪ Fatigue > 39 (clinically significant fatigue)^b^	31 (46.3%)	
▪ Fatigue < 39 (no clinical fatigue)^b^	36 (56.7%)	
**Sleep evaluation**		
Insomnia Severity Index (ISI) score	8.88 ± 6.35	
Pittsburgh Sleep Quality Index (PSQI) score	8.20 ± 4.33	
**Psychological factors**		
HADS-A	7.18 ± 4.98	
HADS-D	6.67 ± 4.41	

^a^Some variables did not sum up to 67 due to missing data

^b^The score of 39 is the defined thresholds for clinical importance (TCIs) for the EORTC QLQ-C30 Computer Adaptive Testing Core measure [52, 53]

### Bivariate analyses

Bivariate analyses taking the fatigue score (FA score) as the dependent variable showed that patients with metastases, particularly bone metastases, exhibited higher fatigue (mean score 80 ± 18.26 for bone metastases versus 38.70 ± 31.39 for the absence of metastases). Moreover, patients receiving palliative chemotherapy and those treated with a capecitabine-based regimen had higher fatigue scores (Table 2). When exploring the continuous variables, results have shown that patients with a lower blood cell count (hemoglobin, leukocytes, and platelets) had significantly higher fatigue scores. Finally, the higher the cycle number, the higher the fatigue score (*p* = 0.007). Pain was not significantly associated with the fatigue level (*p* = 0.124) (Table 3).

**Table 2:** FA score, sociodemographic characteristics, and risk factors

Characteristic (*n* = 67)	Mean (SD)	Mean rank	*p*-value
**Nationality**			
Lebanese (*n* = 60)	41.11 (32.24)	33.40	0.452
Non-Lebanese (*n* = 7)	50.79 (31.98)	39.14	
**Marital status**			
Non married (*n* = 9)	35.80 (32.29)	30.22	0.524
Married (*n* = 58)	43.10 (32.24)	34.59	
**Education level**			
Primary (*n* = 9)	28.40 (24.29)	24.89	0.097
Secondary (*n* = 41)	48.51 (31.70)	36.76	
University (*n* = 15)	34.07 (34.24)	27.60	
**Socioeconomic level**			
Low (*n* = 3)	18.52 (16.97)	18.83	0.177
Middle (*n* = 58)	44.44 (32.58)	35.07	
High (*n* = 5)	24.44 (26.53)	24.10	
**Professional activity**			
Yes (*n* = 22)	48.48 (34.55)	37.55	0.289
No (*n* = 45)	39.01 (30.76)	32.27	
**Alcohol**			
No (*n* = 59)	43.13 (31.36)	34.64	0.460
Yes (*n* = 8)	34.72 (38.69)	29.31	
**Tobacco**			
No (*n* = 45)	45.43 (31.14)	36.01	0.353
Yes (*n* = 19)	37.42 (33.58)	31.11	
Previous smoker (*n* = 3)	22.22 (38.49)	22.17	
**Allergy**			
No (*n* = 62)	39.96 (31.46)	32.77	0.065
Yes (*n* = 5)	68.89 (30.83)	49.20	
**Dyslipidemia**			
No (*n* = 56)	40.08 (31.90)	32.85	0.266
Yes (*n* = 11)	52.53 (32.62)	39.86	
**Diabetes**			
No (*n* = 62)	42.11 (33.19)	33.89	0.865
Yes (*n* = 5)	42.22 (14.49)	35.40	
**Hypertension**			
No (*n* = 50)	44.00 (32.53)	35.20	0.379
Yes (*n* = 17)	36.60 (31.12)	30.47	
**Oral antidiabetics**			
No (*n* = 62)	42.11 (33.19)	33.89	0.865
Yes (*n* = 5)	42.22 (14.49)	35.40	
**Antihypertensive drugs**			
No (*n* = 52)	42.52 (32.76)	34.31	0.806
Yes (*n* = 15)	40.74 (30.77)	32.93	
**Dyslipidemia treatment**			
No (*n* = 57)	39.38 (32.06)	32.40	0.103
Yes (*n* = 10)	57.78 (39.07)	43.10	
**Antidepressant treatment**			
No (*n* = 59)	41.05 (31.17)	33.47	0.542
Yes (*n* = 8)	50.00 (39.84)	37.88	
**Antipsychotic treatment**			
No (*n* = 63)	40.56 (30.80)	33.21	0.178
Yes (*n* = 4)	66.67 (47.14)	46.50	
**Neurogenic pain treatment**			
No (*n* = 64)	40.45 (31.14)	33.16	0.095
Yes (*n* = 3)	77.78 (38.49)	52.00	
**Notable treatment**^a^			
No (*n* = 60)	40.86 (32.52)	32.26	0.122
Yes (*n* = 7)	58.73 (21.96)	43.93	
**Metastasis**			
No (*n* = 60)	38.70 (31.39)	31.93	**0.010**
Yes^b^ (*n* = 7)	71.43 (23.00)	51.71	
**Metastasis type**			**0.021**
No (*n* = 60)	38.70 (31.39)	31.93	Ref
Bone (*n* = 5)	80.00 (18.26)	56.30	0.011
Pulmonary (*n* = 2)	50.00 (23.57)	40.25	0.945
**Chemotherapy type**			**0.005**
Palliative (*n* = 7)	71.43 (23.00)	50.71	Ref
Adjuvant (*n* = 44)	34.63 (31.16)	28.47	0.010
Neoadjuvant (*n* = 16)	52.08 (28.60)	39.50	0.332
**Cyclophosphamide treatment**^c^			
No (*n* = 36)	49.38 (31.02)	38.13	0.057
Yes (*n* = 31)	33.69 (31.75)	29.21	
**Capecitabine treatment**^c^			
No (*n* = 62)	39.07 (31.09)	32.20	**0.007**
Yes (*n* = 5)	80.00 (18.26)	56.30	

Numbers in bold are significant results (*p* < 0.05)

^a^These treatments include benzodiazepines (alprazolam, bromazepam, clonazepam, lorazepam), opioids (tramadol and codeine) and zolpidem

^b^Patients with metastasis were not considered as having a relapsed breast cancer (thus not excluded) because they had a primary diagnosis of metastatic breast cancer

^c^Other treatment types did not give significant results

**Table 3:** Correlation between FA score and continuous variables

	Age	Weight	Height	BMI	BSA	Creatinine	Hemoglobin	Leukocytes	Platelets	Cycle number	VAS score
Correlation with FA score	0.100	0.118	0.180	0.011	0.165	−0.006	**−0.335**	**− 0.297**	**−0.319**	**0.329**	0.190
*p*-value	0.421	0.343	0.144	0.933	0.182	0.963	**0.007**	**0.017**	**0.010**	**0.007**	0.124

Numbers in bold are statistically significant *p*-values; All other variables not mentioned in this table showed a *p* > 0.15 for dependent variables in the bivariate analysis

Neither genetic factors (Table 4) nor sleep and mental scales (Table 5) were significantly associated with the fatigue score.

**Table 4:** Fatigue and genetic characteristics

Characteristic (*n* = 67)	Mean (SD)	Mean rank	*p*-value
***ABCB1*** **rs1045642**			
CC (*n* = 11)	36.36 (26.80)	30.45	0.572
CT (*n* = 27)	34.29 (34.29)	35.87	
TT (*n* = 27)	38.68 (32.67)	31.17	
***COMT*** **rs4680**			
VV (*n* = 18)	42.59 (35.80)	33.06	0.814
VM (*n* = 32)	28.77 (28.77)	32.45	
MM (*n* = 16)	47.92 (36.00)	36.09	
***COMT*** **rs4680** (VV & VM) vs MM			
MM (*n* = 16)	47.92 (36.00)	36.09	0.527
VV & VM (*n* = 50)	40.67 (31.15)	32.67	
***OPRM1*** **rs1799971**			
AA (*n* = 52)	43.16 (31.40)	34.07	0.637
AG (*n* = 14)	39.68 (36.39)	31.39	
GG (*n* = 0)	–	–	
***CLOCK*** **rs1801260**			
TT (*n* = 24)	42.59 (33.20)	28.71	0.933
TC (*n* = 32)	43.75 (30.26)	28.34	
CC (*n* = 0)	–	–	
***PER2*** **rs934945**			
GG (*n* = 40)	37.78 (32.79)	30.69	0.318
GA (*n* = 23)	48.79 (30.47)	37.61	
AA (*n* = 3)	55.56 (38.49)	39.50	
***PER2*** **rs934945** (GG & GA) vs AA			
GG & GA (*n* = 63)	41.80 (32.16)	33.21	0.572
AA (*n* = 3)	55.56 (38.49)	39.50	
***PER2*** **rs934945** GG vs (GA & AA)			
GA & AA (*n* = 26)	30.67 (30.67)	37.83	0.132
GG (*n* = 40)	32.79 (32.79)	30.69	
***CRY2*** **rs10838524**			
GG (*n* = 22)	44.44 (31.80)	35.18	0.564
AG (*n* = 31)	44.09 (32.26)	34.39	
AA (*n* = 13)	35.04 (34.50)	28.54	
***CRY2*** **rs10838524** (GG & GA) vs AA			
AA (*n* = 13)	35.04 (34.50)	28.54	0.289
GG & GA (*n* = 53)	44.23 (31.76)	34.72	
***DRD2*** **rs6277**			
CC (*n* = 9)	59.26 (31.43)	40.72	0.128
CT (*n* = 24)	42.59 (31.03)	31.67	
TT (*n* = 28)	34.52 (31.77)	27.30	
***DRD2*** **rs6277** (CC & CT) vs TT			
TT (*n* = 28)	34.52 (31.77)	27.30	0.126
CC & CT (*n* = 33)	47.14 (31.55)	34.14

**Table 5:** Sleep and mental scales correlations with FA score

	ISI scale	PSQI scale	HADS-A	HADS-D
Correlation with FA scale	0.105	0.078	0.102	0.084
*p*-value	0.402	0.532	0.410	0.500

*Abbreviations*: *ISI* Insomnia severity index, *PSQI* Pittsburgh Sleep Quality Index, *HADS-A* HADS anxiety subscale, *HADS-D* HADS depression subscale

### Multivariable analysis

The multivariable analysis, taking the dichotomized fatigue score as the dependent variable, showed that a higher cycle number and a lower hemoglobin level were significantly associated with higher odds of exhibiting fatigue (ORa of 1.51 and 0.67, respectively). As for genetic factors, our results have shown that having at least one C allele for *DRD2* SNP (CC and CT) was significantly associated with 4.09 higher odds of expressing fatigue compared to TT patients (*p* = 0.047). The *CLOCK* SNP tended toward significance: patients with the TT genotype had lower risks of expressing fatigue than TC patients (Table 6).

**Table 6:** Multivariable analysis using logistic regressions

Factor	ORa	95% CI	*p*-value
**Model 1: Cycle number**			
Cycle number	1.44	1.12–1.85	0.004
**Model 2: Cycle number plus chemotherapy type and factors**			
Cycle number	1.51	1.07–2.12	0.018
Chemotherapy type			0.254
Adjuvant vs Neoadjuvant	0.29	0.02–3.55	0.333
Palliative vs Neoadjuvant	0.77	0.05–10.95	0.844
Capecitabine	767 × 10^6^	0 - Indefinite	1.000
Cyclophosphamide	1.80	0.46–7.01	0.400
Neuropathic pain treatment	1.18	0.08–18.03	0.904
**Model 3**: **Biological measures**			
Hemoglobin	0.67	0.45–0.99	0.046
Leucocytes	1.00	1.00–1.00	0.189
Platelets	1.00	1.00–1.00	0.733
**Model 4: Genes**			
*ABCB1* rs1045642	0.92	0.40–2.12	0.848
*CLOCK* rs1801260 TT vs TC	0.29	0.07–1.15	0.077
*COMT* rs4680 (V/V & V/M vs M/M)	1.37	0.29–6.38	0.693
*PER2* rs934945 (GG & GA) vs AA	0.56	0.02–13.02	0.716
*CRY2* rs10838524 (GG & GA) vs AA	3.12	0.44–21.89	0.253
*DRD2* rs6277 (CC & CT vs TT)	4.09	1.02–16.48	0.047
**Model 5: Full factor model**^a^			
Cycle number	1.36	0.98–1.89	0.066
*CLOCK* rs1801260 TT vs TC	0.29	0.07–1.29	0.104
*DRD2* rs6277 (CC & CT vs TT)	3.79	0.84–17.20	0.084
Hemoglobin	0.38	0.50–1.30	0.376

^a^Factors with *p*-value < 0.10 from other models were introduced

## Discussion

Breast cancer patients experience several long-term physical complications related to chemotherapy, including pain, lymphedema, and fatigue [2, 54, 55]. Despite being one of the most harmful conditions on health-related QOL (damaging outcomes on prognosis, psychosocial, and physical function, e.g., functional disability, social isolation, depression) [56–58], CRF is often overlooked, mainly because it is not correctly and timely evaluated. Identifying the triggers for CRF is paramount for implementing patient-tailored strategies for prevention and early detection [2, 7]. This study aimed to assess fatigue severity and associated factors in a sample of patients with breast cancer.

Our results showed that almost half of our patients reported clinically significant fatigue, with a mean fatigue score of 42.12 ± 32.10, similar to what was previously reported in other breast cancer populations using the same scale (43.92 ± 27.43 and 42.2 ± 30.9) [59–61].

Regarding the genetic aspect, our study revealed novel significant correlations between fatigue and genetic factors, particularly *DRD2* rs6277 and *CLOCK* rs1801260. It could demonstrate that patients who carry at least one C allele (CC and CT) for the c.957C > T (rs6277) of *DRD2* were four times more likely to develop fatigue than TT patients. This SNP affects *DRD2* mRNA stability, thereby influencing the expression of dopaminergic receptors D2 in the brain [62], particularly in the striatal, thalamic, and neocortical areas [63], with a possible consequence on dopamine’s signal transduction [25]. The few studies that examined the effect of rs6277 on physical fatigue have not addressed cancer populations. One research exploring the effects of nicotine on newly exposed individuals has shown that men with TT genotype expressed a decreased fatigue compared to those with CT/CC genotypes [64]. The exact mechanism explaining this observation is yet to be explored.

Another genetic factor that tended toward significance was the SNP of *CLOCK*: patients with TC genotype for the rs1801260 of *CLOCK* had higher risks of exhibiting fatigue than TT patients. To the best of our knowledge, this study is the first to demonstrate a direct association between this polymorphism and CRF in breast cancer patients. However, previous research among non-cancer patients could correlate the C-allele in the SNP rs1801260 of *CLOCK* with eveningness, leading to lower morning physical activity [33]. The SNP c.3111 T > C is located in the 3′-untranslated region; it modifies sleep homeostasis by altering the patient’s biological clock, resulting in abnormalities in physiological processes and sleep-wake cycles [65, 66]. Since fatigue is biologically regulated by sleep-wake homeostasis, sleep disruptions can be a potential risk factor for fatigue. Thus, our results are consistent with previous findings showing an association between fatigue and circadian rhythm disruptions [67, 68].

The study of the biological and clinical factors revealed that a lower hemoglobin level is associated with higher odds of expressing fatigue. Hematological toxicities are a considerable challenge in breast cancer management. They can be related to many factors, including chemotherapy-induced bone marrow suppression [69], nutritional deficiency, vomiting, and cancer/chemotherapy-induced inflammatory syndrome. Inflammatory cytokines produced in cancer patients lead to the upregulation of hepcidin, a protein that blocks the release of iron to transferrin (iron transporter) [70, 71]. This process results in anemia, with a reduced erythropoietic response to anemia and decreased oxygen transfer to tissues and muscles [72], explaining the subsequent fatigue. Therefore, in fatigue management, clinicians should highlight the importance of assessing correctly and treating anemia, whether by blood transfusions or erythropoietin (mainly epoetin alfa) as stated by international guidelines (American Society of Clinical Oncology (ASCO) and European Society for Medical Oncology (ESMO) [73, 74]). Such an approach considerably improves hemoglobin levels and quality of life during chemotherapy among breast cancer patients [75–77].

Finally, our results revealed that the risk of fatigue significantly increased with the number of chemotherapy cycles, in agreement with previous findings showing that breast cancer patients undergoing chemotherapy reported more fatigue over time compared to baseline [78–81]. This fatigue is consequent to cumulative factors precipitating CRF, e.g., chemotherapy [82], pain, depression, anxiety, emotional distress, sleep disturbance, cachexia, anemia [83, 84].

### Limitations and strengths

Our study has several limitations, especially related to the small sample size for genetic analyses and the absence of baseline evaluation (first chemotherapy cycle) since patients could have exhibited fatigue even before starting chemotherapy regimens. Moreover, some modifiable determinants that could significantly influence fatigue were not reported, such as physical activity and nutritional status (even if the BMI can be a surrogate measure of nutrition status [3, 85]). Indeed, several studies reported that a good nutritional status and high physical functioning improve HRQoL in breast cancer patients [3, 85–87]. Finally, although fatigue was evaluated with a tool validated in cancer patients (EORTC QLQ-C30), the use of other extensive and specific scales, such as the recently Arabic validated EORTC QLQ-BR23 [88], the Functional Assessment of Chronic Illness Therapy-Fatigue (FACIT-F) [89], or the Fatigue Inventory-20 (MFI-20) [90] would have allowed a better evaluation of all fatigue effects and aspects.

However, despite the small sample size, multivariable analyses identified several genetic factors that have been rarely explored for their association with fatigue.

Future longitudinal studies, using a larger sample and more specific scales to evaluate fatigue, are needed to confirm our preliminary findings and explore their potential translation into clinical practice.

## Conclusions

Our study showed that anemic breast cancer patients with a high number of chemotherapy cycles and those carrying at least one C allele for *DRD2* and *CLOCK* SNPs are at greater risk of exhibiting fatigue. Since no previous research has reported such genetic results, future studies are necessary to confirm our findings, allowing clinicians to prioritize the management of patients at higher risks of fatigue during chemotherapy and tailor physical/psychological/cognitive-behavioral interventions to mitigate CRF while improving the quality of life of patients and their families.

## Data Availability

The datasets generated and/or analyzed during the current study are not publicly available due to the fact that the study is still ongoing on other cancer populations (other than breast cancer), but are available from the corresponding author on reasonable request.

## Abbreviations

ASCO: American Society of Clinical Oncology
BBB: blood-brain barrier
BMI: Body mass index
BSA: Body Surface Area
CBC: Complete blood count
ClCr: Creatinine clearance
*CLOCK*: Circadian Locomotor Output Cycles Kaput gene
COMT: Catechol-O-methyl transferase (protein)
*COMT*: Catechol-O-methyl transferase gene
CRF: Cancer-related fatigue
*CRY2*: Cryptochrome circadian Regulator 2 gene
*DRD2*: dopamine receptor D2 gene
EORTC QLQ-C30: The European Organization for Research and Treatment of Cancer quality of life questionnaire
ESMO: European Society for Medical Oncology
FA: Fatigue
FACIT: Functional Assessment of Chronic Illness Therapy system of Quality of Life questionnaires
FACIT-F: Functional Assessment of Chronic Illness Therapy - Fatigue
HADS-A: Hospital Anxiety and Depression Scale; anxiety subscale
HADS-D: Hospital Anxiety and Depression Scale; depression subscale
HDF: Hôtel-Dieu de France
HPA: hypothalamic-pituitary-adrenal
HRQoL: Health-related quality of life
ISI: Insomnia Severity Index
MFI-20: Fatigue Inventory-20
MOR: μ-opioid receptor
P-gp: P-glycoprotein
*PER2*: Period 2 gene
PSQI: Pittsburgh Sleep Quality Index
QOL: Quality of life
SNP: Single nucleotide polymorphism
TCI: thresholds for clinical importance
VAS: Visual analogue scale
